# Beyond pharmacotherapy: a narrative review of patient, social, and healthcare system factors influencing treatment adherence in ankylosing spondylitis

**DOI:** 10.3389/fmed.2026.1888657

**Published:** 2026-07-27

**Authors:** Yao Wang, Lifei Feng, Xiaoqiu Zhu, Miaomiao Zhang

**Affiliations:** Department of Nursing, Sir Run Run Shaw Hospital, Zhejiang University School of Medicine, Hangzhou, China

**Keywords:** ankylosing spondylitis, digital health, exercise adherence, rehabilitation, shared decision-making, treatment adherence

## Abstract

Ankylosing spondylitis (AS) is a chronic inflammatory rheumatic disease characterized by inflammatory low back pain, new bone formation, and progressive ossification of the axial skeleton. Over time, it causes irreversible structural damage to the spine and sacroiliac joints, profoundly impairing patients’ quality of life and physical function. Without timely and effective intervention, AS can lead to permanent functional disability. Evidence suggests that biological, psychological, and social factors jointly undermine treatment adherence, with complex interactions among these dimensions that make single-domain interventions insufficient. Improving adherence to treatment regimens therefore requires a thorough understanding of these multifaceted influences and the development of evidence-based, multicomponent strategies. This narrative review synthesizes literature published from 2010 to 2024, drawn from PubMed/MEDLINE, Embase, Web of Science, the Cochrane Library, and CNKI, to identify factors contributing to non-adherence in AS patients, with particular attention to disease knowledge, perceptions of treatment efficacy, exercise and rehabilitation adherence, and social support. Wherever possible, evidence derived directly from AS or axial spondyloarthritis (axSpA) populations is distinguished from findings extrapolated from broader rheumatology or chronic-disease literature. Based on this synthesis, targeted recommendations for optimizing treatment management and enhancing long-term patient outcomes are proposed, together with priority directions for future AS-specific adherence research.

## 1 Introduction

Ankylosing spondylitis is a chronic inflammatory disease primarily affecting the axial skeleton. Its hallmark features—inflammatory low back pain, bony ankylosis, and spinal new bone formation—lead to progressive loss of spinal mobility, physical limitation, and a substantial decline in quality of life as the disease advances. Diagnosis is established according to the modified New York criteria. A notable challenge is the diagnostic delay of 5–10 years that typically elapses between symptom onset and confirmed diagnosis, underscoring the urgency of early recognition and adherence-focused education.

Successful AS management depends to a considerable degree on sustained patient engagement with long-term treatment plans, encompassing regular follow-up visits and consistent adherence to prescribed regimens. Poor adherence is associated with higher disease activity, worsening symptoms, and deteriorating overall health. Biological agents (BAs) have been shown to meaningfully improve health-related quality of life (HRQoL) in AS patients, which underscores the connection between good adherence and favorable clinical outcomes (1). Moreover, when patients actively participate in treatment decisions and are well-informed about the efficacy and safety profiles of BAs, adherence tends to improve substantially.

The determinants of treatment adherence in AS are multidimensional. Psychological conditions such as anxiety and depression are closely linked to reduced adherence (2); socioeconomic constraints, including limited income and restricted access to healthcare, present additional barriers; illness perception and disease-related stigma add to patients’ psychological burden and frequently discourage care-seeking. Conversely, adequate disease knowledge promotes adherence by encouraging patients to take their health more seriously, while effective support from healthcare professionals and constructive patient–provider communication further reinforce adherence behaviors (3).

**FIGURE 1:**
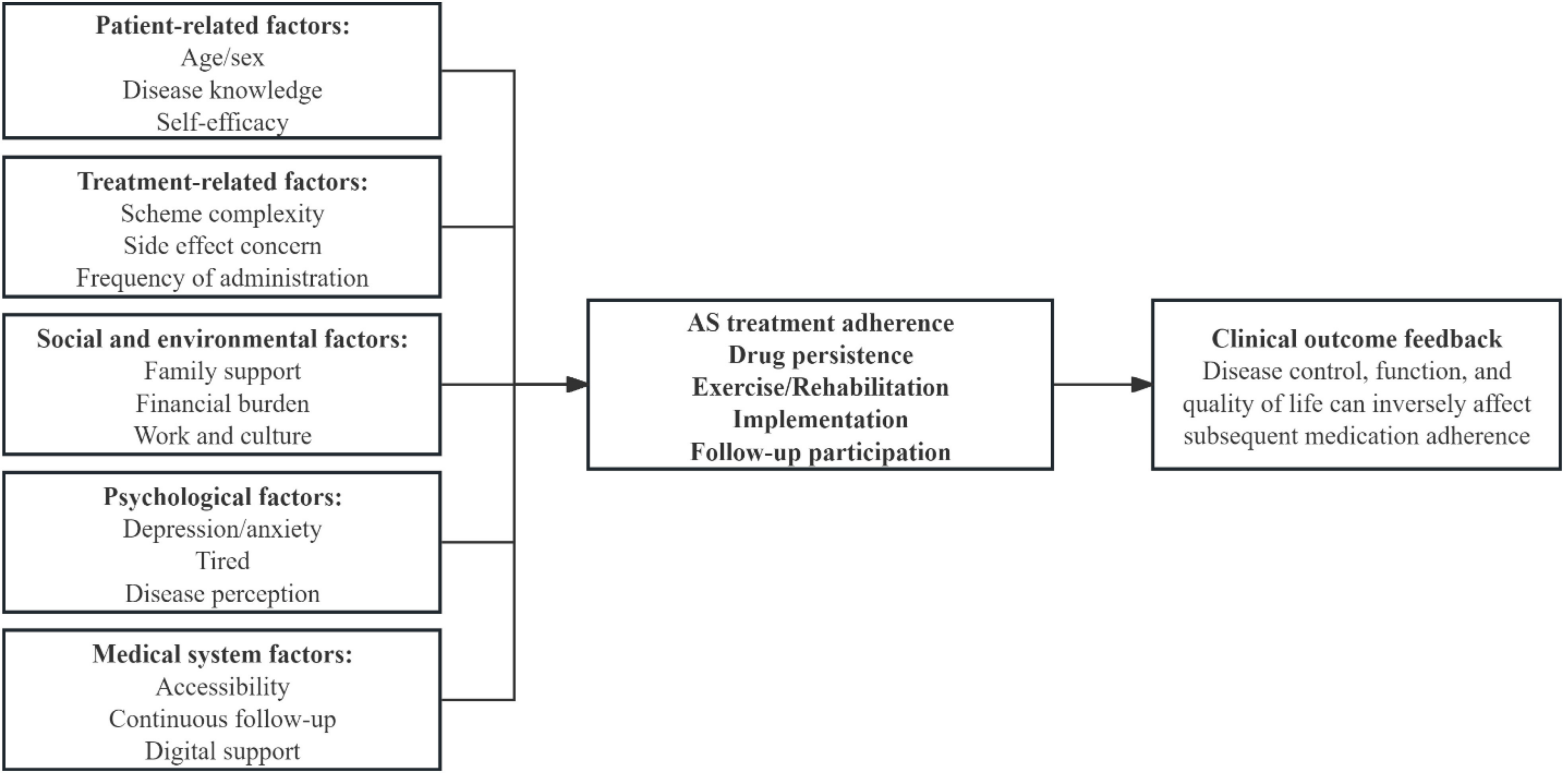
A multidimensional framework of factors influencing reatment adherence in ankylosing spondylitis. Patient-related, treatment-related, social and environmental, psychological, and healthcare system factors jointly act on AS treatment adherence, which in turn feeds back through disease control, function, and quality of life. (arrows denote clinically plausible interactions and feedback relationships rather than established single-factor causal effects.).

Examining these influences in depth is essential for improving clinical outcomes and advancing evidence-based practice. Employing a narrative integrative review approach, this review draws on published literature from 2010 to 2024 to elucidate the barriers and facilitators that shape patients’ ability to follow clinical guidelines. Existing literature is marked by notable methodological limitations—particularly regarding non-pharmacological interventions, standardization of adherence measurement, and the degree of patient involvement—all of which represent important gaps (4). By integrating findings across multiple studies, this review aims to provide a comprehensive picture of the current state of adherence in AS and to offer actionable improvement strategies that may inform clinical management and enhance patient satisfaction (Figure 1).

This review is explicitly conceived as a narrative integrative review rather than a systematic review or meta-analysis. Relevant literature was identified through structured searches of PubMed/MEDLINE, Embase, Web of Science, the Cochrane Library, and CNKI for records published between 1 January 2010 and 31 December 2024, supplemented by hand-searching the reference lists of clinical guidelines, systematic reviews, and key primary studies. Core search concepts combined terms for “ankylosing spondylitis/axial spondyloarthritis,” “adherence/compliance/persistence,” “exercise/rehabilitation/physiotherapy,” “medication,” and “digital health/telehealth.”

Priority was given to studies conducted in AS or axSpA populations, together with clinical guidelines, systematic reviews and meta-analyses, randomized controlled trials, observational studies, and qualitative research addressing barriers to adherence. Evidence originating from other rheumatic or chronic diseases is used only as background or mechanistic context and is not treated as directly equivalent to AS-specific findings; this distinction is made explicit throughout the review and is summarized in Table 1. In interpreting the evidence, particular attention was paid to differences across studies in patient population, definition of adherence, measurement instruments, follow-up duration, and outcome metrics, all of which limit direct comparability between reported adherence rates.

**TABLE 1:** Key determinants of treatment adherence in ankylosing spondylitis: evidence sources, limitations, and potential intervention strategies.

Factor domain	Evidence source	Main limitations	Potential intervention strategies
Patient-related factors	Relatively abundant evidence from AS/axSpA populations; additional support from exercise-adherence and mental-health literature	Gaps in disease knowledge, low self-efficacy, and biased perceptions of illness severity or treatment risk	Disease education, shared decision-making, goal-setting, and self-management support
Psychological factors	AS/axSpA studies support associations between depression/anxiety and disease activity/quality of life	Predominantly cross-sectional design; causal direction requires longitudinal confirmation	PHQ-9/GAD-7 screening, psychological support, sleep and fatigue management
Treatment-related factors	Medication persistence, dosing convenience, and side-effect concerns are supported by dedicated studies	Pharmacy-refill or self-reported adherence may not fully reflect actual behavior	Regimen simplification, proactive side-effect management, clear communication about expected efficacy and discontinuation risk
Exercise/rehabilitation factors	Substantial variability in reported adherence to AS exercise regimens; supervised, home-based, and remote programs have all been studied	Evidence is stronger for short-term efficacy than for long-term adherence; marked heterogeneity across intervention formats	Short-term supervised initiation, home-based consolidation, periodic feedback, remote/digital support
Social and healthcare-system factors	AS-specific direct evidence is comparatively limited, though effects of employment, cost, transportation, and access are well-documented	Substantial cross-country variation in healthcare-system structure limits extrapolation	Continuity of follow-up, flexible scheduling, linkage to rehabilitation resources, supportive policy

## 2 Patient-related factors

Treatment adherence in AS is shaped by a range of patient-level characteristics, including illness perception, motivation for treatment, and comorbidity burden. Research indicates that patients with more severe disease tend to demonstrate higher adherence to exercise programs, suggesting that symptom burden may in fact act as a driver of active engagement (5). Self-efficacy is positively associated with adherence; patients who feel capable of managing their condition are more likely to sustain treatment over the long term (6). Educational interventions that help patients understand the importance of lifestyle modification can consequently improve overall adherence. Age and educational background also matter: younger patients tend to prefer group-based therapy, whereas older patients typically favor individualized approaches.

### 2.1 Demographic variables (age, sex, and related factors)

Demographic characteristics such as age and sex are important determinants of adherence in AS. Older patients may differ substantially from younger counterparts in their understanding of and adaptability to treatment regimens; those with longer disease duration often report a greater perceived need for medication (7). The accumulation of comorbidities in older patients—including osteoporosis and cardiovascular disease—further complicates treatment decision-making and adherence management. Sex differences are also clinically relevant: male and female patients may respond differently to physical therapy. A study examining treatment preferences among patients with axial spondyloarthritis (axSpA) found a strong preference for individualized approaches across the sample, suggesting that sex may influence the choice of treatment modality. These differences collectively argue for the clinical importance of tailoring AS management strategies to individual patient profiles.

### 2.2 Psychological factors (depression and anxiety)

Chronic conditions such as AS exact a psychological toll that is no less significant than their physical effects, with depression and anxiety representing the most prevalent mental health concerns. AS patients consistently report higher levels of both conditions compared with the general population, and these elevated levels adversely affect adherence and overall health outcomes. Evidence confirms a meaningful association between disease severity and psychological wellbeing (8). Of particular note, depression undermines adherence through two distinct mechanisms: it reduces patients’ motivation to attend appointments (an indirect effect) and leads directly to medication-skipping and discontinuation of exercise. Patients with persistently elevated disease activity, as reflected by high Bath Ankylosing Spondylitis Disease Activity Index (BASDAI) scores, experience the greatest psychological burden (9). Routine psychological screening using validated instruments such as the PHQ-9 is therefore recommended in clinical practice. Applying an integrative biopsychosocial framework can help clinicians navigate the complex interplay between physical symptoms and mental health, thereby supporting sustained patient engagement with treatment.

### 2.3 Illness perception and disease knowledge

An accurate understanding of AS is a prerequisite for sustained treatment engagement. The extent to which patients grasp what their disease entails directly shapes both their perception of their treatment plan and their broader health management behaviors. One study found that more than 75% of AS patients were unaware of the relationship between their condition and an elevated cardiovascular risk, revealing a substantial knowledge deficit (10). Beyond cardiovascular risk, patients frequently lack awareness of the risks of osteoporosis, uveitis complications, and the association between intestinal inflammation and AS—underscoring the need for patient education to address the full spectrum of AS’s multisystem involvement. Insufficient knowledge of non-pharmacological therapies has been identified as a significant barrier to guideline adherence, pointing to an urgent need for targeted training of both patients and clinicians (11). Educational interventions directed at these knowledge gaps represent a high-priority strategy for improving adherence and optimizing disease management outcomes.

### 2.4 Perceived disease severity

Patients’ perception of their disease severity exerts a substantial, though bidirectional, influence on treatment adherence. Individuals typically assess severity by integrating symptom burden, functional limitation, and emotional distress. Those who regard their condition as more serious generally display stronger motivation to adhere, both to exercise programs and to pharmacological therapy. Systematic reviews have confirmed that greater disease severity and longer diagnostic delay are associated with improved adherence to recommended exercise regimens (5). However, overemphasizing severity can backfire: some patients respond with helplessness and nihilism, abandoning treatment as a result. Clinical communication must therefore strike a careful balance between conveying the seriousness of the disease and fostering realistic expectations. On the other side, patients who believe their condition is well-controlled may underestimate the need for ongoing treatment, neglecting long-term management and compromising future outcomes. Gaining insight into individual patients’ illness perceptions is thus essential when designing effective adherence-promotion strategies.

### 2.5 Personal beliefs and attitudes toward treatment

Patients’ beliefs about treatment and their attitudes toward medication are important psychological determinants of adherence. The degree to which a patient perceives medication as necessary directly influences their willingness to persist with it. Research shows that while most patients recognize the benefits of pharmacological treatment, concerns about side effects are a principal adherence barrier (based on the specific subscale of the Beliefs about Medicines Questionnaire). Systematic reviews further reveal that fear of self-injection (needle phobia) and cultural biases against medication dependence are commonly underestimated barriers that clinicians should proactively identify and address. Patients with longer disease duration tend to hold stronger beliefs in the necessity of treatment and thus report higher adherence motivation (7, 12). Patients’ subjective assessment of risks associated with different treatment options may also influence their decision-making. For these reasons, eliciting and addressing patients’ individual beliefs and attitudes toward treatment is central to improving adherence in AS.

## 3 Treatment-related factors

Developing effective treatment strategies for AS requires careful consideration of treatment-related factors that affect adherence. Reported adherence rates vary markedly across studies—ranging from 51% to 95%—a variability that largely reflects heterogeneity in how adherence is measured and reported (12). Physical therapists have found that strategies such as individualized exercise programs and setting realistic short-term goals help sustain patient engagement. Breaking adherence objectives into concrete, near-term action steps tends to support more durable behavioral change than broad, long-term goals alone.

### 3.1 Treatment regimen complexity

The chronic, fluctuating nature of AS creates an inherently complex treatment landscape. Patients must balance medication adherence with recommended physical activity and lifestyle modifications, and evidence confirms that adherence is dynamic rather than static, shifting in response to changes in disease status and treatment regimen (13). Studies show a notable decline in adherence when patients are required to take medication more than twice daily, suggesting that switching to long-acting biologics administered weekly or monthly is a practical strategy for improving adherence through regimen simplification. In addition, patients may develop individualized exercise routines that do not fully align with clinical recommendations as they independently assess their own health (14), further complicating adherence management. This tension between medical guidance and personal health management reinforces the value of individualized intervention strategies.

### 3.2 Medication side effects and tolerability

Adverse drug reactions and individual tolerability are pivotal determinants of medication adherence in AS. The experience of side effects may lead patients to discontinue treatment unilaterally, destabilizing disease management. Although biological disease-modifying antirheumatic drugs (bDMARDs) offer well-established clinical benefits, concerns about their adverse effects remain an important adherence challenge. Research has found that a considerable proportion of AS patients continue to use opioid analgesics even while receiving newer, target-to-remission therapies (15), suggesting that current pain management strategies need further refinement. Clinicians should discuss expected side effects and management plans with patients before initiating treatment and establish regular follow-up contacts to enable early detection of and timely response to side effect–driven non-adherence. Improving tolerability through supportive care and clear communication about side effect management has been shown to enhance long-term adherence and quality of life in AS (16).

### 3.3 Access to treatment options

The availability of diverse treatment options is an important systemic factor shaping adherence in AS. Evidence points to structural barriers within both pharmacological and physical therapy pathways that impede patients’ ability to follow clinical guidelines (17). These barriers typically stem from inadequate knowledge of available resources, poor patient–provider communication, and financial constraints. The expanding availability of telemedicine and digital health platforms offers a promising avenue for reducing these access gaps, particularly for patients in remote areas or those with mobility limitations. As patients develop individualized management strategies to cope with dynamic disease states (18), the centrality of patient education and healthcare professional support in facilitating adherence becomes all the more evident.

### 3.4 Treatment duration and dosing frequency

Both the duration of treatment and the frequency of dosing have far-reaching effects on medication adherence in AS. Because AS requires lifelong management, patients’ understanding of how long they need to take medication and how often directly shapes their appraisal of treatment necessity and their willingness to accept the associated burdens. With increasing treatment duration, many patients develop a heightened sense of medication dependence, a perception especially pronounced among those with longer disease histories. Polypharmacy intensifies concerns about side effects and regimen complexity, both of which can compromise adherence behavior (19). Treatment fatigue is a well-recognized phenomenon in chronic disease management, and AS patients are particularly prone to self-reducing or stopping medication during asymptomatic remission periods. Clinicians should therefore regularly reassess patients’ treatment motivation at routine visits and provide timely, individualized re-engagement interventions. Individualized regimens that take both treatment duration and dosing frequency into account offer one of the most practical approaches to sustaining long-term adherence in AS.

### 3.5 Patient–provider communication

High-quality communication between patients and their healthcare providers is indispensable for maintaining adherence to AS treatment plans. The condition presents longstanding challenges of prolonged diagnostic delay and complex management strategies. Primary care physicians have noted that patients are often reluctant to report back pain for a variety of reasons, and this reticence can lead to symptom underestimation and increased management complexity (20). Systematic reviews similarly highlight numerous barriers to adherence with published treatment guidelines. Building a relationship of trust and clearly establishing treatment expectations are key communication strategies that encourage patients to voice their concerns openly, enabling timely clinical intervention and improved adherence.

### 3.6 Exercise and rehabilitation adherence

Exercise and rehabilitation are central to the long-term management of AS (21), yet knowing that exercise is necessary is not the same as being able to sustain a prescribed exercise program over time. Available evidence suggests that adherence to exercise in AS/axSpA is jointly shaped by disease activity, pain, fatigue, working hours, access to rehabilitation resources, and self-efficacy; reported adherence rates vary considerably across studies, largely because exercise prescriptions, definitions of adherence, and follow-up periods differ substantially from one study to another (5, 18). Clinical discussion of exercise adherence should therefore move beyond a binary “exercises or does not exercise” framing to consider frequency, duration, movement quality, safety, and long-term maintenance separately.

Supervised exercise programs can improve initial engagement through movement correction, immediate feedback, and safety monitoring, whereas home-based programs are more easily integrated into daily life but rely more heavily on patients’ own self-management capacity. Group-based or aquatic exercise may enhance patients’ sense of support and participation, while telerehabilitation and mobile health tools can help reduce barriers related to time and distance (22). However, high-quality, AS-specific evidence on which delivery format best sustains long-term adherence remains limited (23), and no single model can be assumed to suit all patients.

A pragmatic strategy is a hybrid model that combines brief supervised initiation, an individualized home-based program, and periodic feedback and adjustment: clinicians first assess disease activity, functional limitations, pain triggers, and patient preference; patients and clinicians then jointly set achievable short-term goals; and progress and symptom changes are subsequently tracked through clinic visits, telephone contact, electronic patient-reported outcomes (ePROs), or mobile applications. For patients with persistently poor long-term adherence, the priority should be identifying the specific barrier involved rather than simply increasing the frequency of education.

## 4 Social and environmental factors

Social and environmental factors constitute an important external dimension of AS treatment adherence. Family support, income, and community resources all influence patients’ ability to meet the demands of their treatment. Restricted access to physical therapy—a cornerstone of AS management—directly diminishes treatment effectiveness. One study noted that despite receiving physical therapy, a substantial proportion of axSpA patients participated only in passive individual therapy lacking active exercise components (24), reflecting a systemic quality gap. Growing evidence supports the role of social determinants of health in AS adherence, suggesting that healthcare systems should incorporate social prescribing into comprehensive AS management frameworks.

### 4.1 Support from family and friends

Family members and close friends play an irreplaceable role in AS management, and the support they provide has a profound influence on both treatment adherence and overall wellbeing. Emotional support helps patients cope with the psychological burden of chronic disease and strengthens their capacity for self-management. Research confirms a close association between AS disease status and psychological problems such as anxiety and depression (25), indicating that a strong social support network can buffer against these difficulties. The bidirectional relationship between disease activity and psychological health (26) further underscores how important it is for patients to feel genuinely understood and supported by those around them. Encouraging open communication and authentic expressions of concern from family and friends is an effective means of motivating patients to sustain treatment and improving both health outcomes and quality of life.

### 4.2 Socioeconomic status and financial barriers

Treatment adherence in AS is shaped not only by medical factors but also by patients’ socioeconomic status (SES). Financial hardship is a primary barrier to accessing healthcare services such as physical therapy, directly limiting patients’ capacity to follow recommended exercise regimens. Patients from socially disadvantaged backgrounds consistently demonstrate lower adherence rates (27). More specifically, the high cost of biologic therapies is the leading driver of cost-related non-adherence; expanding patient assistance programs and improving access to biosimilars are important policy levers for addressing this problem. AS patients also frequently conduct informal cost–benefit analyses when deciding whether to participate in exercise activities, reflecting the pervasive influence of financial concerns on health behavior.

### 4.3 Cultural influences on health behavior

Culture plays a far-reaching role in shaping health behaviors and adherence to treatment in chronic disease. When managing their condition, AS patients engage in an ongoing cognitive weighing of personal circumstances analogous to a cost–benefit analysis, a process deeply colored by cultural context. In certain cultural settings, traditional norms of “enduring illness,” distrust of modern medicine, or preferences for alternative healing practices may lead patients to delay care, discontinue medication, or seek alternative treatments. Healthcare teams must be culturally competent to recognize and effectively respond to these barriers. Patients with higher disease activity typically report greater anxiety and depression (28), and the interaction between psychological state and cultural background further modulates treatment adherence.

### 4.4 Availability of healthcare resources

The availability of healthcare resources is a critical structural determinant of treatment adherence in AS. Adequate access to specialist care—including rheumatologists—is essential for effective long-term disease management. However, barriers to guideline adherence are pervasive; in the United States, for example, the number of studies specifically evaluating guideline adherence in AS remains very small (29). The availability of physical therapy, rehabilitation programs, and patient education services substantially affects the extent to which patients can engage with their treatment plan (30). An imbalanced ratio of rheumatologists to patients is a global challenge. Adopting a multidisciplinary team (MDT) model—integrating rheumatology, physical therapy, psychology, and social work—represents a viable and effective response to this shortfall. A shortage of healthcare resources can also amplify patients’ frustration and helplessness, further eroding adherence to recommended treatments.

### 4.5 Work and lifestyle influences on treatment

The relationship between occupational life, lifestyle, and AS treatment adherence is bidirectional and multifactorial. Employment provides financial security and a sense of purpose, but it can also introduce workplace stress and reduce the time available for the physical activity that AS management requires (31). AS patients who maintain regular exercise and a healthy lifestyle consistently demonstrate better health outcomes and higher quality of life than those who are physically inactive (22). Workplace accommodations—such as flexible working hours and height-adjustable workstations—have been shown to reduce work-related pain aggravation, thereby indirectly strengthening adherence to treatment regimens. Incorporating work–life balance strategies alongside lifestyle modifications into individualized treatment plans holds considerable practical value for improving overall adherence in AS.

## 5 Healthcare system factors

Healthcare system factors interact with AS treatment adherence in complex ways and are constrained by numerous systemic challenges. One of the core problems in axSpA/AS management is inconsistent adherence to clinical guidelines. Evidence suggests that many healthcare providers are unable to implement guidelines effectively in practice owing to resource and support constraints, which compromises both disease activity monitoring and the systematic recommendation of comprehensive treatment approaches, including non-pharmacological therapies. Embedding disease activity score prompting tools into electronic health record (EHR) systems—for example, automated BASDAI/ASDAS calculation modules—has the potential to standardize clinical follow-up workflows and reduce management inconsistencies caused by missed assessments.

### 5.1 Quality of care provided by healthcare professionals

The quality of care delivered by healthcare professionals has a decisive influence on treatment adherence in chronic conditions such as AS. High-quality communication between rheumatologists and patients forms the foundation of trust and helps patients understand both the content of their treatment plan and the rationale for sustained engagement. Educational intervention programs have been shown to improve adherence in spondyloarthritis patients by motivating active participation in their own health management. At a practical level, providing patients with a written, personalized summary of their treatment plan at the conclusion of each clinical encounter can substantially reduce recall errors and improve real-world adherence. Addressing systemic shortcomings—such as insufficient disease activity monitoring and inadequate recommendation of non-pharmacological therapies—can further raise the quality of care and improve adherence outcomes.

### 5.2 Continuity of care and follow-up management

Strong continuity of care and a robust follow-up strategy are fundamental to AS management and jointly shape both adherence and overall outcomes. Patients who navigate complex healthcare systems and attend irregular appointments in primary care settings are at risk of fragmented care and symptom misattribution. Lack of care coordination can compound the fluctuating nature of AS symptoms, making it harder for patients to sustain consistent adherence to both treatment and exercise regimens (32). Comprehensive, coherent care has been shown to enhance patients’ sense of being supported and understood, which in turn reinforces adherence (33). Employing a dedicated AS nurse specialist to conduct telephone follow-ups and provide patient education is a cost-effective model for improving care continuity that has been implemented and shown to work well in several European countries.

### 5.3 Patient education and counseling practices

High-quality patient education and counseling underpin the entire adherence enterprise in AS. Evidence indicates that adherence to recommended exercise programs remains low overall, despite the critical role of physical activity in disease management. Understanding the complex interplay among patients’ perceptions of exercise, their beliefs about disease management, and their decision-making processes is key to improving engagement (23). Digital patient education platforms—such as AS-specific mobile applications and online health management tools—can deliver personalized, real-time knowledge, and when combined with regular online interactive sessions, they can simultaneously improve disease literacy and self-management confidence. Targeted educational approaches that address the specific barriers to both medication and exercise adherence are necessary components of any effective adherence intervention (34).

### 5.4 Insurance coverage and the policy environment

Health insurance serves as an important institutional mechanism for sustaining patients’ commitment to their treatment plans. Insurance policies influence access to care and, through cost-sharing arrangements, shape patients’ decisions about whether to seek recommended services. When coverage for biologic therapies and physical therapy is unstable, patients may be compelled to interrupt critical treatments because of limited financial capacity. Published literature specifically examining how insurance-related payment barriers affect treatment adherence in AS remains limited; existing evidence from broader rheumatology adherence reviews suggests that out-of-pocket costs, co-payment, and insurance type may influence biologic adherence (35), while AS-specific evidence on insurance coverage and treatment patterns remains underdeveloped (36). Studies in inflammatory arthritis more broadly have documented the real difficulties patients face in obtaining timely diagnosis and treatment, reinforcing the need for a policy environment that facilitates early access to care and shortens waiting times.

### 5.5 The role of technology in treatment management

Digital technology has demonstrated considerable promise for improving treatment adherence and optimizing healthcare communication in AS. Mobile health applications and telemedicine have become important tools for maintaining patient engagement with treatment and enabling more individualized care plans. Technology is particularly well-suited to addressing gaps in guideline adherence; review evidence confirms that novel technological solutions can meaningfully reduce adherence barriers in practice (37). Wearable devices—such as smart bands and motion sensors—offer an additional advantage by objectively quantifying daily physical activity in AS patients. These devices provide clinicians with adherence data beyond what self-report alone can capture and can motivate patients through real-time feedback. As technology continues to evolve, its application in AS management has the potential not only to enhance adherence but also to create a supportive environment that accompanies patients throughout their entire treatment journey.

## 6 Conclusion

A comprehensive understanding of the multidimensional factors that influence treatment adherence in AS is a prerequisite for improving clinical outcomes and healthcare efficiency. This review demonstrates that psychological and social factors—including disease knowledge, patient–provider communication, and social support systems—are central elements of any effective adherence intervention. Targeted biological therapies have achieved notable advances by acting on specific cytokine signaling pathways (38). Yet these pharmacological advances do not automatically translate into improved adherence; without simultaneously addressing behavioral and psychological barriers, even the most sophisticated agents cannot fully realize their therapeutic potential. This recognition makes the case that adherence interventions must evolve in tandem with drug development.

**FIGURE 2:**
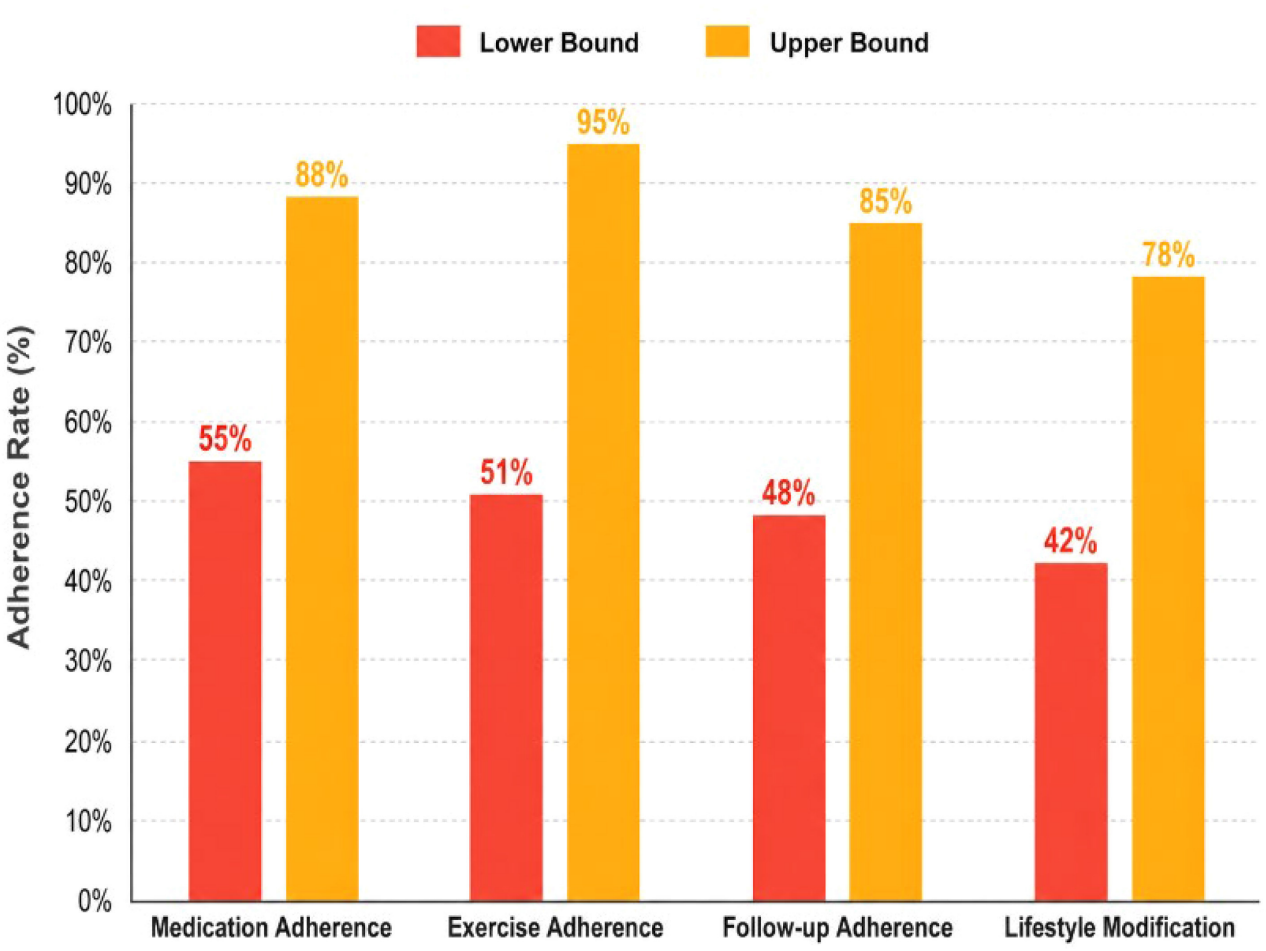
Summary of adherence rate ranges across treatment modalities in ankylosing spondylitis. Red bars represent the lower bound and green bars the upper bound of adherence rates reported in individual studies, reflecting the marked heterogeneity in current adherence measurement and the pressing need for standardized instruments.

The evidence base reveals extreme variability in adherence to exercise regimens across AS populations, with reported rates ranging from 51.4% to 95% (Figure 2) (39). This range should not be interpreted as a direct comparison of adherence across different treatment categories, since the underlying studies differ substantially in patient population, definition of adherence, measurement instruments, and follow-up duration; rather, it underscores the need for standardized adherence measurement tools that separately report medication persistence, exercise/rehabilitation completion, and follow-up attendance. This degree of heterogeneity points to an urgent need for standardized adherence measurement instruments. Educational interventions have been shown to promote adherence, and both greater disease severity and longer time to diagnosis are associated with higher rates of exercise adherence. Research on preferences for biologic treatment indicates that efficacy, safety, and convenience of administration—including self-injection capability and reduced dosing frequency—are the primary considerations for patients and rheumatologists alike when making treatment decisions (40).

Integrating patient-reported outcomes (PROs) into routine AS clinical care is an important step toward improved adherence. AS has been shown to impair HRQoL across multiple dimensions, and meaningful outcome improvement requires addressing both the physical and emotional aspects of disease simultaneously (41). Collecting PRO data—using instruments such as BASDAI, BASFI, and SF-36—at every clinic visit and feeding back visualized results to patients is recommended as a means of reinforcing their perception of treatment progress and consolidating adherence behavior. A shared decision-making framework that draws patients into treatment discussions, and that acknowledges and respects their individual preferences regarding medication efficacy, safety, and mode of administration, can substantially strengthen patients’ sense of therapeutic agency and their motivation to adhere (42).

A multidisciplinary approach is the cornerstone of adherence improvement in AS. Combining the expertise of rheumatologists, psychologists, and physical therapists enables the development of comprehensive treatment plans tailored to each patient’s unique needs. Research demonstrates that anxiety and depression are typically associated with higher disease activity in AS (43), underscoring the clinical necessity of addressing both the somatic and psychological dimensions of the disease concurrently. Priority areas for future research should include: (1) developing and validating AS-specific adherence measurement tools; (2) evaluating the long-term effects of digital health interventions; (3) exploring culture-specific adherence barriers and targeted responses; and (4) conducting randomized controlled trials based on MDT models (Table 2).

**TABLE 2:** Specific priority directions for future research on treatment adherence in ankylosing spondylitis.

Research direction	Specific focus
AS-specific adherence instruments	Separately measuring medication, exercise/rehabilitation, and follow-up adherence, with reliability, validity, and cross-cultural validation
Longitudinal cohort studies	Clarifying the temporal relationships among disease activity, psychological status, treatment beliefs, and adherence
Rehabilitation adherence intervention trials	Comparing the long-term execution rates, functional outcomes, and cost-effectiveness of supervised, home-based, group-based, and remote rehabilitation
Digital health evaluation	Assessing the long-term effectiveness, privacy and safety, usability, and digital equity of mHealth tools, ePROs, and wearable devices
Multidisciplinary care models	Evaluating the impact of collaborative rheumatology–rehabilitation–psychology–nursing models on adherence and quality of life

In summary, improving treatment adherence in AS requires a multicomponent approach that integrates several complementary strategies. Establishing a trusting therapeutic relationship is fundamental, as it creates the psychological safety for patients to openly express their concerns and preferences. Patient education that strengthens disease literacy and self-management skills can effectively dispel the cognitive distortions and psychological fears that impede adherence. Digital tools such as mobile health applications help sustain engagement with treatment plans through reminder functions and progress tracking. Ultimately, a patient-centered adherence framework—one that integrates disease education, psychological support, technological enablement, and policy safeguards—will be the defining element of optimized long-term AS management.

Accordingly, the recommendations offered in this review fall into two categories. Patient education, regular exercise, shared decision-making, and continuous disease-activity monitoring are supported by existing AS/axSpA guidelines, systematic reviews, or reasonably consistent primary studies, and can therefore be regarded as evidence-based recommendations. Telerehabilitation, digital monitoring, and multidisciplinary collaborative models, by contrast, are evidence-informed implementation strategies whose long-term benefit for adherence still requires confirmation in AS-specific, longitudinal, and comparative studies.

## 7 Clinical implications

This review provides a comprehensive analysis of the multidimensional factors shaping AS treatment adherence—encompassing patient psychology, treatment complexity, and social determinants—and proposes a systematic intervention framework grounded in the biopsychosocial model. It emphasizes patient-centered care, including routine incorporation of patient-reported outcomes and the adoption of shared decision-making, as the core of adherence-promotion efforts, and recommends motivational interviewing and individualized education as first-line adherence interventions. The review further maps key research gaps and advocates for a multidisciplinary integrative approach to AS adherence, calling on future investigations to prioritize digital health tools, culturally sensitive interventions, and the standardization of adherence measurement.
